# Atypical Guillain–Barre syndrome with T6 sensory level

**DOI:** 10.1002/ccr3.6414

**Published:** 2022-10-08

**Authors:** Osamah Al‐Ameen, Mohanad Faisal, Salma Mustafa, Mohammed Alhatou

**Affiliations:** ^1^ Department of Medical Education Hamad Medical Corporation Doha Qatar; ^2^ Neurology Department Hamad Medical Corporation Doha Qatar

**Keywords:** acute inflammatory demyelinating polyneuropathy, acute motor sensory axonal neuropathy, Guillain barre syndrome, neurology, sensory level

## Abstract

Guillain–Barré syndrome is an acute immune‐mediated demyelinating disease. Typical features include progressive ascending lower extremity weakness and areflexia. Several variants have been described that can make the diagnosis challenging. Here, we report a case of GBS presenting with progressive lower limb weakness and T6 sensory level.

## Abstract 2

To bring attention to the wide spectrum of presentation of Guillain–Barre syndrome by presenting a rare, atypical variant with a sensory level which was successfully managed by our team so that clinicians keep this deferential in mind when they face patients with such neurological manifestation.

## 1 INTRODUCTION

Guillain–Barre syndrome (GBS) is an acute immune‐mediated inflammatory demyelinating polyneuropathy, and it is the most common cause of acute flaccid paralysis worldwide.

It is occurring with an overall incidence of 1–2 cases per 100,000 people per year. The disease's incidence rises by about 20% every decade of age, and males are more likely to contract it than females.^1^

It mainly affects most of the spinal nerve roots and peripheral nerves and often involves the cranial nerves and is characterized by symmetrical limb weakness and areflexia.^2^

Most patients present with an antecedent illness, most commonly an upper respiratory tract infection or diarrheal illness, before the onset of progressive motor weakness. Several microorganisms have been associated with Guillain–Barré syndrome, most notably Campylobacter jejuni, Zika virus, influenza, Mycoplasma pneumoniae, and cytomegalovirus and in 2020, the severe acute respiratory syndrome coronavirus.^3^

Diagnostic criteria for GBS were originally proposed for research in 1978 by the National Institute of Neurological Disorders and Stroke (NINDS). Including, acute progressive and symmetric muscle weakness with absent or depressed deep tendon reflexes.^4^ Brighton criteria can also be used for diagnosis, and it has four levels of diagnostic certainty.^5^ It can also be used in resource‐limited settings. In a study conducted in India, Levels 1, 2, and 3 detected 62%,84%, and 86% of the cases of GBS, respectively.^6^

The weakness can vary from mild difficulty with walking to nearly complete paralysis of all extremities, including facial, respiratory, and bulbar muscles. Symptoms typically progress over days to 4 weeks. Patients may also have mild sensory symptoms and dysautonomia.

Lumbar puncture for cerebrospinal fluid analysis should be performed in all patients to confirm the GBS diagnosis and exclude other sources of the symptoms. With a typical finding of an elevated cerebrospinal fluid analysis (CSF) protein with a normal white blood cell count.

Electrodiagnostic studies consisting of nerve conduction studies (NCS) and electromyography (EMG) are performed in most patients to support the diagnosis of GBS as well as to provide prognostic information regarding the nature and severity of nerve dysfunction.

Multiple variants of GBS have been described. Here, we will present a rare case of GBS that presented with lower limb weakness, areflexia, and atypical sensory level.

## 2 CASE PRESENTATION

A 61‐year‐old Indian female with a past medical history of diabetes mellitus and chronic mechanical back pain presented with progressively worsening lower limb weakness, gait difficulty, and reduced sensation in the abdomen and lower limbs, and numbness in her trunk and bilateral lower extremities. She denied urinary or bowel incontinence. There were no abnormal movements or a reduced level of consciousness. Her symptoms were preceded by a febrile watery diarrheal illness 7 days before the onset of these symptoms. However, on presentation, her diarrhea had already subsided. Family and social history were unremarkable. Her vital signs were within the normal range. Physical examination showed intact consciousness, speech, and cranial nerves. Lower limb power was 3/5 proximally and 4/5 distally. Upper limb power was normal. Reflexes were 1+ in the upper limb and areflexia in the lower limb. The sensory examination showed loss of sensation in all modalities, including light touch, pain, temperature, joint position, and vibration below the T6 level**.** Upper limb sensation was normal. Finger‐to‐nose and heel‐to‐shin tests were normal, but gait was unsteady.

**FIGURE 1:**
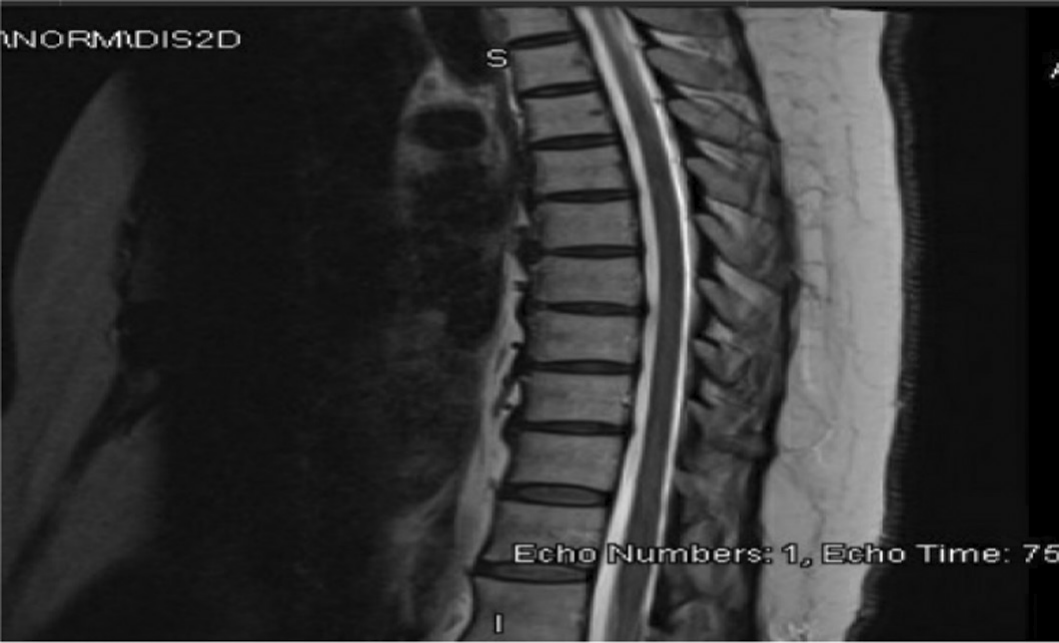
MRI of the thoracic spine

The initial workup showed leukocytosis (WBC of 12.000) with a normal complete metabolic profile. Urgent magnetic resonance images (MRI) of the whole spine with contrast (Figure 1) to exclude transverse myelitis or cord compression were inconclusive apart from mild multilevel degenerative changes seen in the mid‐cervical, mid‐dorsal, and lower lumbar regions, predominantly at the level of C5‐C6, C6‐C7, T8‐T9, L3‐L4, and L4‐L5. CSF studies showed protein of 1.57 gm/dl with normal cell counts consistent with albumin‐cytologic dissociation (Table 1). CSF tests for infection were unremarkable as well, including bacterial and virologic studies. Nerve conduction studies showed absent F‐waves of both perineal and tibial nerves, otherwise an unremarkable study. Needle examination revealed positive sharp waves with decreased recruitment in both iliopsoases, consistent with early neurogenic changes. Denervation was also seen in the L1‐L2 paraspinal muscles.

**TABLE 1:** Cerebrospinal fluid analysis

CSF Analysis		Normal Values
Appearance	Clear	Clear
Color	Colorless	Colorless
RBC	333/uL	(0–2)
WBC	8/uL	(0–5)
CSF Glucose	7.49 mmol/L	(2.22–3.89)
CSF Protein	1.57 gm/L	(0.15–0.45)
Albumin Cytologic Dissociation	Present	Absent
CSF Culture	Negative	

In view of her clinical presentation of acute flaccid paralysis with areflexia, unremarkable MRI findings, CSF albumin‐cytologic dissociation, and absent lower extremity F‐waves, she was diagnosed with having possible GBS and she was started on a 5 days course of intravenous (IV) immunoglobulin with a standard dose of 0.4 gm/kg, which was later followed by a 5 day course of IV pulse methylprednisolone. At first, her lower limb weakness worsened to 1/5 and she developed bowel and bladder dysfunction.

A follow‐up MRI of the spine 1 week after the first one did not show any new changes. Over the next 2–4 weeks, the patient started to recover gradually, with improvement in her sensations in the first 2 weeks, followed by recovery of motor symptoms in 4 weeks with the help of an occupational therapist and physiotherapist. On follow‐up 4 months later, she remained independent in all activities of daily living.

## 3 DISCUSSION

GBS manifests itself in a variety of ways, ranging from typical peripheral 4 limb weakness with areflexia to numerous variants that complicate diagnosis and management. Typical GBS usually presents with ascending limb weakness with hyporeflexia/areflexia with minor sensory symptoms and usually involves the 4 upper limbs.^4^ Besides the typical presentation of GBS, clinical variants are based on the types of nerve fibers involved (motor, sensory, sensory, and motor, cranial, or autonomic) and the mode of fiber injury (demyelinating vs axonal).^7^, ^8^ The most common mode of nerve fiber injury is demyelinating, while axonal motor and axonal sensory variants have been described^8^ and are associated with rapid progression and poor prognosis.^9^ Axonal variants are usually preceded by campylobacter jejune infection.^10^

GBS typically has ascending weakness, but descending variants have been described, including the Miller–Fischer variant, which consists of ophthalmoplegia, ataxia, and areflexia.^11^ Another cranial variant is the Bickerstaff brainstem encephalitis, which is characterized by alteration in consciousness, hyperreflexia, ataxia, and ophthalmoplegia.^12^

A less common paraparetic motor variant affects the legs selectively with areflexia, mimicking an acute spinal cord lesion, and is associated with back pain.^13^ In most cases, the paraparetic variant is usually milder compared to the classical GBS, and in 50% of the cases, there are abnormalities in upper limb nerves observed in NCS.^14^

While the sensory symptoms in GBS are typically mild, pure sensory variants have been described and are usually characterized clinically by exclusive sensory symptoms and signs that reach their nadir in a maximum of 6 weeks without related systemic disorders and other diseases or conditions.^15^

Our case is a 62‐year‐old lady who presented with progressive ascending lower limb weakness with areflexia in addition to the loss of all modalities of sensation below the level of T6 with no neurological symptoms and signs in the upper limbs. MRI with contrast excluded spinal cord compression and demyelination. CSF studies showed albumin‐cytological dissociation. NCS showed absent F‐waves of both the perineal and tibial nerves. She was given intravenous immunoglobulin followed by 5 days of IV methylprednisolone. Our most likely diagnosis was GBS based on the presentation of acute flaccid paralysis, areflexia, preceding diarrheal illness, CSF studies, and lack of evidence of demyelination and cord compression on MRI, in addition to the duration and course of her illness following Brighton and NINDS criteria. We think that our case is like the case reported by Khoo et al. and may represent a new variant of GBS.^16^ It is important to note that typical NCS findings are not always present in GBS as it is affected by timing and multiple studies at different times are needed.^17^ The steroid was added to the initial treatment as there was the possibility of transverse myelitis initially as MRI of spine may be normal on acute presentation.^18^ This possibility was later ruled out after a repeat MRI which was normal also. We do not think our patient had transverse myelitis as she does not fulfill the diagnostic criteria^19^ in the absence of demyelination in repeated spine MRI and no CSF leukocytosis.

## 4 CONCLUSION

GBS varies in its presentation, and atypical presentation with sensory level may represent a new variant. Further studies are needed to confirm our suspicion.

## AUTHORS CONTRIBUTIONS

Osamah Al‐Ameen wrote the manuscript, responsible for clinical follow‐up, literature review, and editing. Mohanad Faisal made contributions to writing the manuscript and literature review. Salma Mustafa took part in clinical follow‐up, introduction writing, and reference proofreading. Mohammed Alhatou took part in revising the manuscript and editing it.

## CONFLICT OF INTEREST

The authors have no conflict of interest to declare.

## CONSENT

Written consent has been obtained from the patient according to journal policy.

## Data Availability

The data that support the findings of this study are available from the corresponding author upon reasonable request.
